# Mixed‐phenotype acute leukemia with a predominant B/T and a small subset of myeloid lineage expression

**DOI:** 10.1002/jha2.107

**Published:** 2020-10-16

**Authors:** Maryam Pourabdollah, Entsar Eladl, Aijun Liu, Hong Chang

**Affiliations:** ^1^ Department of Laboratory Haematology Laboratory Medicine Program University Health Network University of Toronto Toronto Ontario Canada; ^2^ Department of Haematology Beijing Chaoyang Hospital Capital Medical University Beijing China; ^3^ Pathology Department Mansoura University Egypt

**FIGURE 1:**
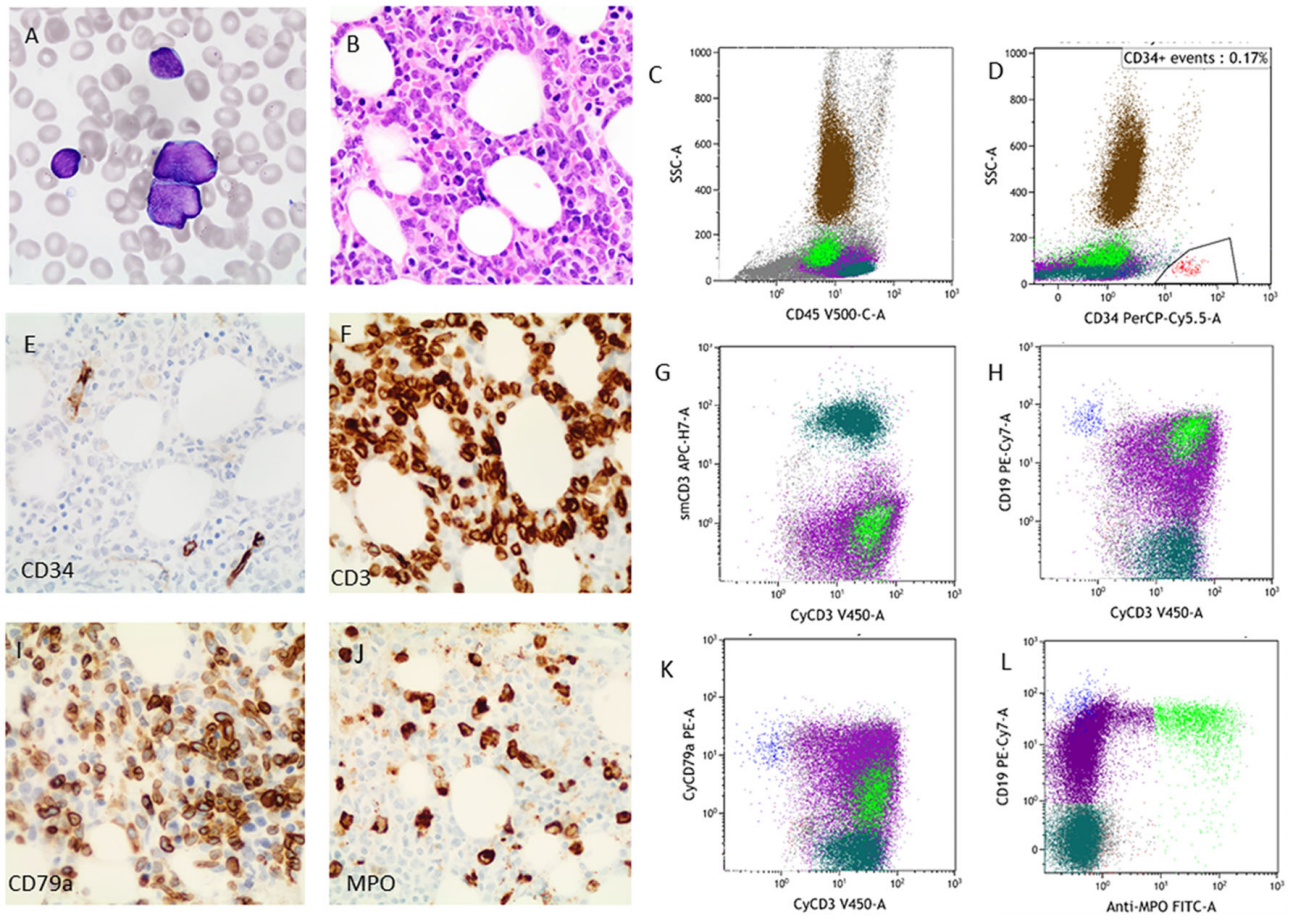
Morphology and immunophenotypic features of the case with MPAL.

A 45‐year‐old man presented with cervical lymphadenopathy. A full blood count showed mild anemia (124 g/L), neutropenia (0.9 × $10^{9}$/L), and thrombocytopenia (115 × $10^{9}$/L). A peripheral blood film demonstrated rare circulating blasts. The bone marrow aspirate was hemodilute with 45% blasts (Figure 1, panel A). The bone marrow biopsy showed decrease in trilineage hematopoiesis and sheets of blasts (Figure 1, panel B). By flow cytometry, the dim CD45 blast population was negative for CD34 but coexpressed cCD3, CD19, and cCD79a (Figure 1, panels C, D, G, H, and K) with a small subset (10% of blasts), and this small subset also expressed myeloperoxidase (panel L) as well as CD13, CD33, and CD117. By immunohistochemistry, the CD34 negative blasts (Figure 1, panel E) were positive for CD3 (panel F), CD79a (panel I), and myeloperoxidase (only in a small subset) (panel J).

Cytogenetics study revealed an abnormal karyotype with multiple structural aberrancies: 46,XY,der(1)t(1;7)(q32,q32),der(3)add(3)(p25)add(3)(q26),der(7)(p15)t(1;7)(q32,q32)[16]/46,XY[4]. Molecular genetic analysis is negative for t(9;22), *KMT2A* or *MECOM* gene rearrangement; no clinically relevant variant was detected on next‐generation sequencing (NGS). The final diagnosis of mixed‐phenotype acute leukemia (MPAL), T/B/myeloid was made. The patient achieved complete remission after treatment with the acute lymphoblastic leukemia targeted, modified Dana‐Farber protocol.

MPAL, T/B/myeloid, is exceptionally rare. Our case satisfies the WHO classification criteria for both T‐and B‐lineage assignment (cyCD3 and CD19/CD79a intensities reach that of normal T‐ and B‐lymphocytes, respectively), and a small subset of blasts also expressed myeloid‐lineage markers. This case has a complex karyotype, but in the absence of the abnormalities that are listed in myelodysplastic syndrome (MDS)‐related cytogenetic changes specified by the WHO classification system (2). Recent studies revealed a spectrum of somatic mutations in MAPL such as *DNMT3A, PHF6*, and mutations in the *JAK‐STAT* pathway. This case is unusual as none of the mutations was detected with our NGS panel of 49 clinically relevant genes including aforementioned genes. Recent evidence on MPAL leukemogenesis suggested that phenotypically different subpopulations of blasts in MPAL share founding genetic lesions with phenotypic plasticity.

